# New approach to improve COP and heat recovery in transcritical CO_2_ refrigeration system for milk processing application

**DOI:** 10.1038/s41598-025-90067-3

**Published:** 2025-02-14

**Authors:** Prosenjit Singha, Chayan Das, Mani Sankar Dasgupta, Souvik Bhattacharyya, Armin Hafner

**Affiliations:** 1 Department of Mechanical Engineering, BITS Pilani, Pilani, 333031 India; 2 TCG CREST, Kolkata, 700091 India; 3 Norwegian University of Science and Technology, Trondheim, 7031 Norway

**Keywords:** CO_2_ transcritical, Subcooling, Parallel compression, Energy efficiency, Evaporative cooling, Engineering, Mechanical engineering

## Abstract

In hot climates, subcooling or after-cooling is an effective method to enhance the coefficient of performance (COP) of CO_2_ transcritical refrigeration system. This study investigates improvement of two contemporary subcooling arrangements: Integrated mechanical subcooling (IMS) and dedicated mechanical subcooling (DMS) and evaporative cooling arrangement to gascooler by introduction of gravity-fed evaporator in a dual evaporator parallel compression system suitable for milk processing. Using location-specific average meteorological data, the performance of the proposed systems is evaluated for Pune, India. Comparative analysis is conducted against a baseline transcritical CO_2_ system with flash gas bypass but lacking any subcooling arrangement. A considerable improvement in COP is observed when subcooling is combined with parallel compression. Incorporation of evaporative cooling with parallel compression yields 62.3% improvement in COP over the flash gas bypass system. However, heat recovery potential is considerably reduced by adopting evaporative cooling. Additionally, the study quantifies a potential reduction in water consumption of 45.6% over a system using flash gas bypass with an indirect evaporative cooling arrangement, and a reduction of 34.3% over a system employing parallel compression with a split gas cooler indirect evaporative cooling arrangement.

## Introduction

The use of synthetic refrigerants in the chlorofluorocarbon (CFC) category, which cause ozone depletion, has mostly been phased out under the global agreement of Montreal Protocol adopted in 1987. Later the Montreal Amendment in 2007 focused on regulating the use of Hydrochlorofluorocarbon (HCFCs), as HCFC molecules released to the atmosphere can eventually reach the stratosphere and decompose due to photolysis and produce ozone depleting CFC^1^. Consequently, hydrofluorocarbons (HFCs) were introduced as non-ozone depleting alternatives to CFCs. However, some of these were found to possess remarkably high global warming potential. Following the footsteps of Montreal Protocol, the Kigali amendment in 2016 proposed a time-bound phase-out of HFCs as well to control global warming. While a relatively new class of synthetic refrigerant, Hydrofluoroolefin (HFOs), having very low GWP and zero ODP, was introduced as a promising alternative^2^. Of late they have been identified as potential source of perfluoroalky and polyfluoroalkyl substances, collectively known as PFAS and are regulated for being bioaccumulative^3^. Need for their phase out and remediation is emphasized by the European Chemicals Agency^4^. It is, therefore, crucial to reassess natural fluids like ammonia, carbon dioxide, hydrocarbons etc., and develop technology to enhance their performance in refrigeration and heat pump application. This approach aims to reduce environmental risks linked with direct release of harmful chemicals to the biosphere.

CO_2_, a natural refrigerant, is gaining popularity in large heating-cooling systems like milk processing and supermarkets due to its superior performance at low ambient temperatures^5,6^. Although the CO_2_ cycle’s COP decreases at high ambient temperatures due to its low critical temperature and high pressure, it remains a clean solution for simultaneous heating and cooling. CO_2_ offers benefits such as high density, thermal conductivity, and low viscosity, making it ideal for compact systems with low flammability and toxicity. To reduce throttling losses in transcritical CO_2_ systems, methods like flash gas bypass (FGB) and parallel compression (PC) are used to improve system performance. Subcooling techniques, including Internal Heat Exchangers (IHX)^7^, Dedicated Mechanical Subcooling (DMS), Integrated Mechanical Subcooling (IMS), economizers, and various mixtures^8^.Further enhance efficiency, with IHX now a standard feature in CO_2_ systems.

Sarkar and Agrawal^9^ explored a CO_2_ transcritical system with PC and economizer and a performance improvement up to 47.3% is reported. In a theoretic study, Llopis et al.^10^ reported that, with DMS, the COP and cooling capacity of a transcritical CO_2_ system can be improved by 20% and 28.8% respectively due to the reduction in heat rejection pressure and improvement in cooling capacity. The authors also stated that the margin of improvement is higher at ambient temperature above 25 °C. While using only DMS with transcritical CO_2_ system^11^, the performance improvement is reported around 30.7%. Further, the advantage of reduction in gascooler pressure for CO_2_ transcritical system is explored by adopting DMS^12^ and resulting possibility of energy savings up to 12% and the reduction of gascooler pressure by 10 bar was claimed. While in an experimental study with IMS and DMS in PC configuration. Andrez et al.^13^ demonstrated an increase in COP in three conditions: 4.1% in IMS and 7.8% in DMS at 25.0 °C, 7.2% for IMS and 13.7% for DMS at 30.4 °C, and 9.5% for IMS and 17.5% for DMS at 35.1 °C. This study supports the argument that as the system’s operating temperature increases, its performance improves with the adoption of subcooling techniques.

Evaporative cooling arrangement can lower the temperature of incoming air and is another possible approach for subcooling at low wet bulb temperature ambient. This is isenthalpic cooling process in which the air temperature is decreased by transferring heat from the air to evaporating water by absorbing required latent heat from the air^14^. Lata and Gupta^15^ presented a comparative study of a booster transcritical CO_2_ system equipped with evaporative cooled gascooler and reported an annual energy saving up to 35%. They also reported performance improvement of about 10–28% for various climatic conditions. An experimental investigation on dual evaporator transcritical CO_2_ systems^16^ at high ambient condition reported that evaporative cooling for supply air to the gascooler can lead to COP improvement of 67.4% at 45 °C ambient.

A contemporary technique showing promise in enhancing system performance is gravity-fed evaporator. It provides improved contact between the liquid refrigerant and the heat exchanger surface area, leading to higher heat transfer rate and hence a more compact heat exchanger design. Hazarika et al.^17^ reported based on experimental study that gravity-fed heat exchangers have more effective heat transfer and the heat transfer coefficient is about double that of a DX heat exchanger. Further experimental investigation by Hafner et al.^18^ reported the performance of a dual-evaporator ejector-based heat pump chiller with a direct expansion (DX) evaporator in the second stage and a gravity-fed evaporator in the first stage. The authors observed a larger temperature drop in the water at the gravity-fed evaporator compared to the DX evaporator. Additionally, the gravity-fed evaporator did not require a pump for refrigerant circulation, unlike a conventional pump-circulated flooded evaporator system, resulting in energy savings.

The above-mentioned studies, demonstrate that both mechanical subcooling and evaporative cooling, among many other methods, are viable solutions to improve the performance of a transcritical CO_2_ system. However, the comparison of performance improvement between these strategies in different ambient conditions are yet to be explored. Though, it is claimed that PC is not advisable with evaporative cooling^19^ but the scenario can differ when the gravity-fed evaporator is adopted. In a gravity-fed evaporator system, flash gas generation in the receiver increases which may lead to greater amount of fluid handled by the auxiliary compressor. The present study endeavors to fill the aforementioned research gap and evaluates the performance of a CO_2_ transcritical system with subcooling and evaporative cooling along with gravity-fed evaporator. The system performance is evaluated in terms of coefficient of performance (COP) and combined coefficient of performance (CCOP). The study also explores water savings in evaporative cooling by using a split gas cooler arrangement Further, in case of milk processing application, both heating and cooling is essential. Evaporative cooling and subcooling strategies effects both heating and cooling performance of the system under the variable ambient condition which is yet to explore. To the best of the authors’ knowledge, it is a maiden attempt to evaluate the system performance for a multi-evaporator milk processing application with demand for both heating and cooling in warm ambient.

### System description

A milk processing systems demands two evaporators, one of them maintained at 0 °C for pasteurization, which is assumed to be gravity fed and another, a DX evaporator maintained at -15 °C for cold room. The cooling capacity of these two evaporators are 50 kW and 150 kW respectively. The evaporator load and temperature is taken from a Pune based milk processing facility. In this study, performance of eight different system configurations are explored to identify the performance suitable for milk processing application when gravity-fed evaporator is consider with high ambient condition.

**Fig. 1:**
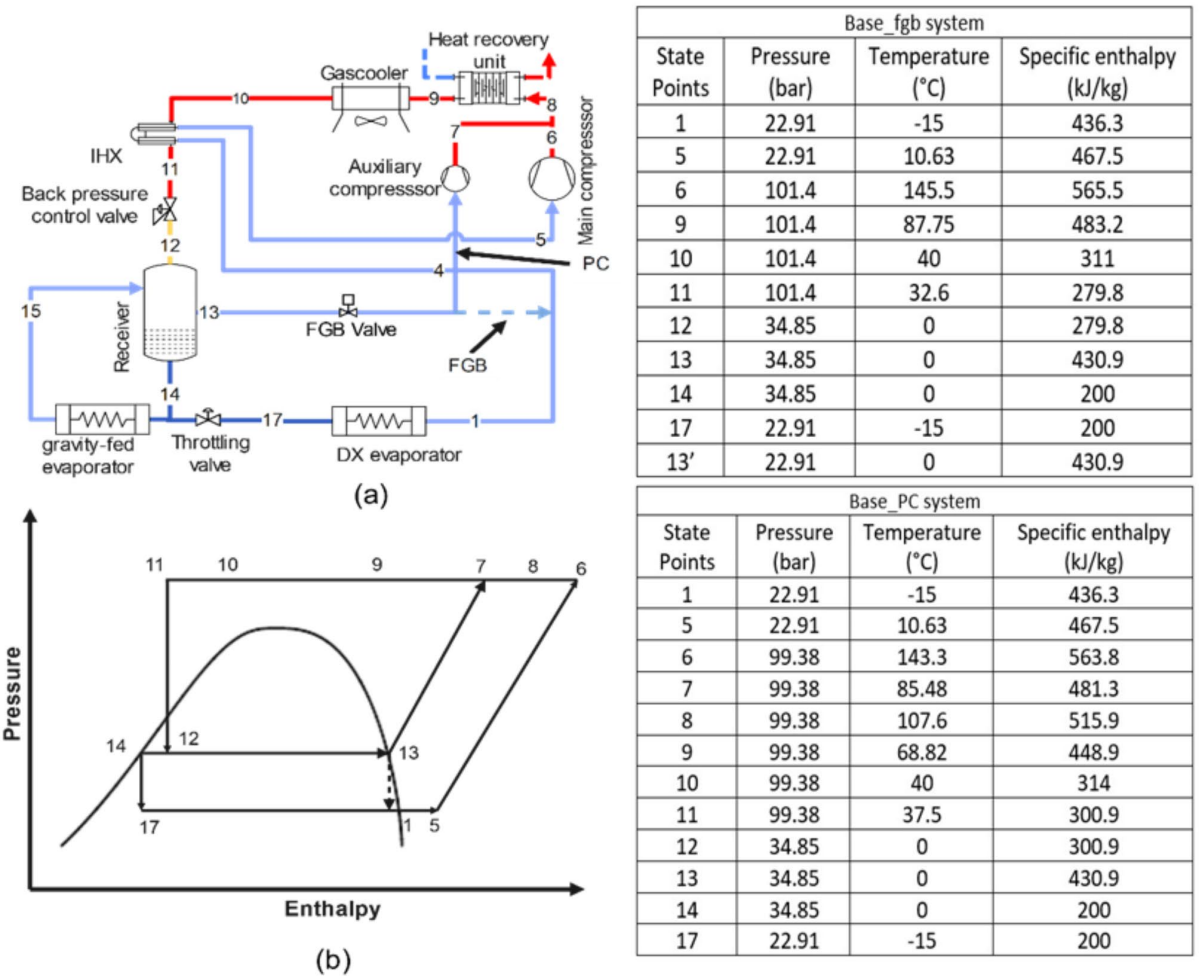
(**a**) Schematic of Base_fgb and Base_PC (**b**) corresponding p-h diagram. Table represents the state points according to P-h diagram of Base_fgb and Base_PC system.

**Fig. 2:**
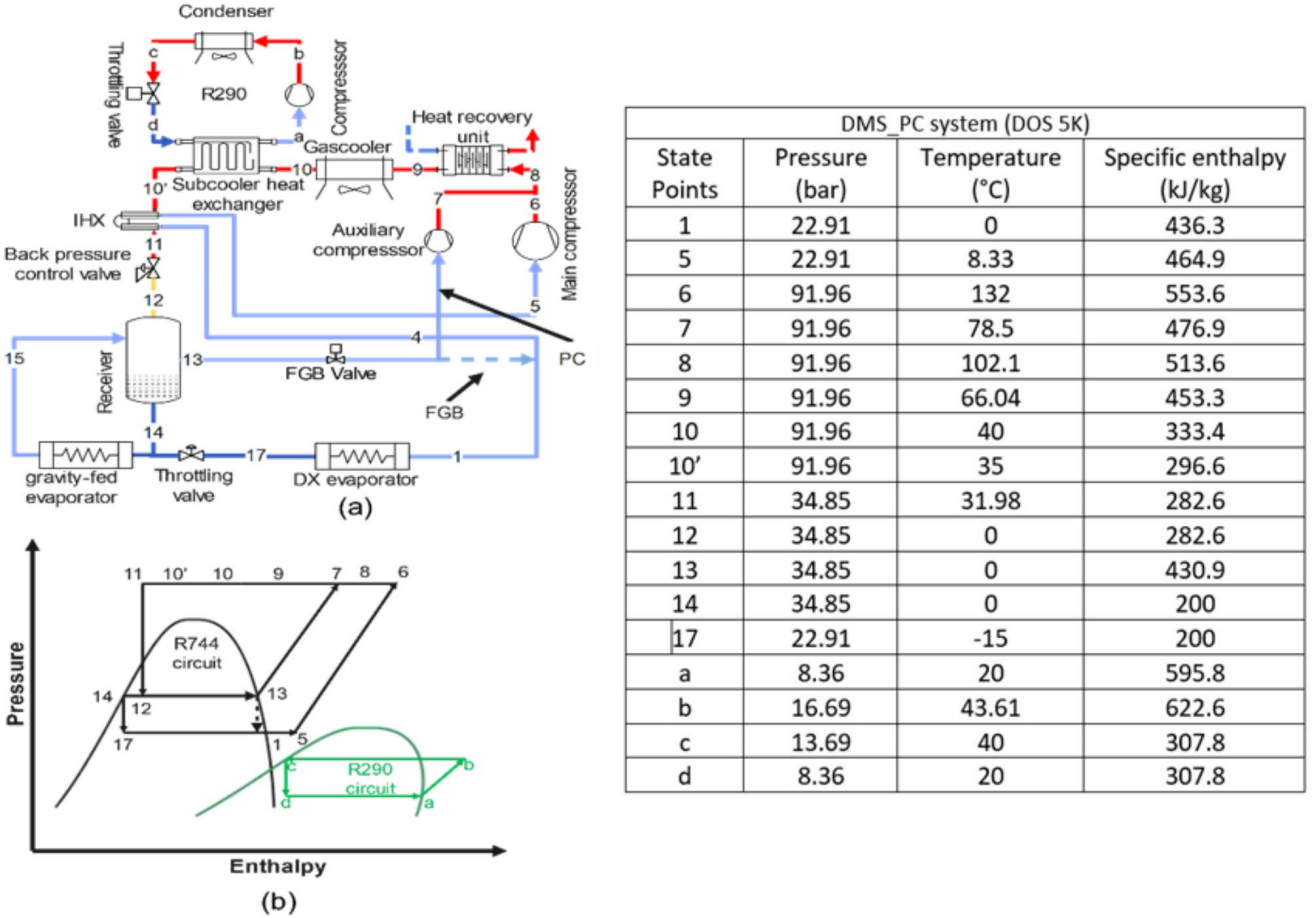
(**a**) Schematic of DMS_fgb and DMS_PC (**b**) corresponding p-h diagram. Table represents the state points according to P-h diagram of DMS_PC system for DOS of 5 K.

**Fig. 3:**
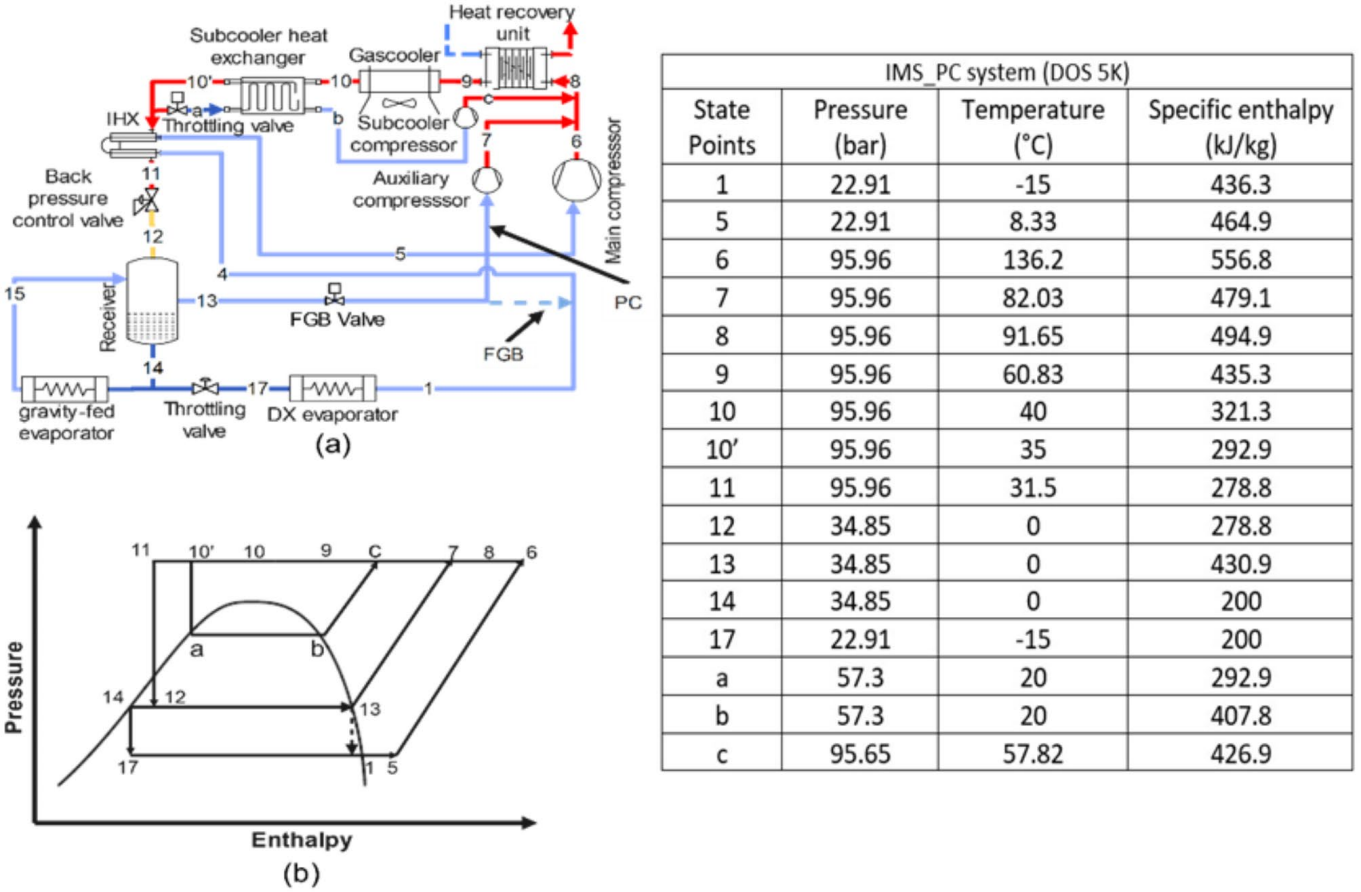
(**a**) Schematic of IMS_fgb and IMS_PC (**b**) corresponding p-h diagram. Table represents the state points according to P-h diagram of IMS_PC system for DOS of 5 K.

**Fig. 4:**
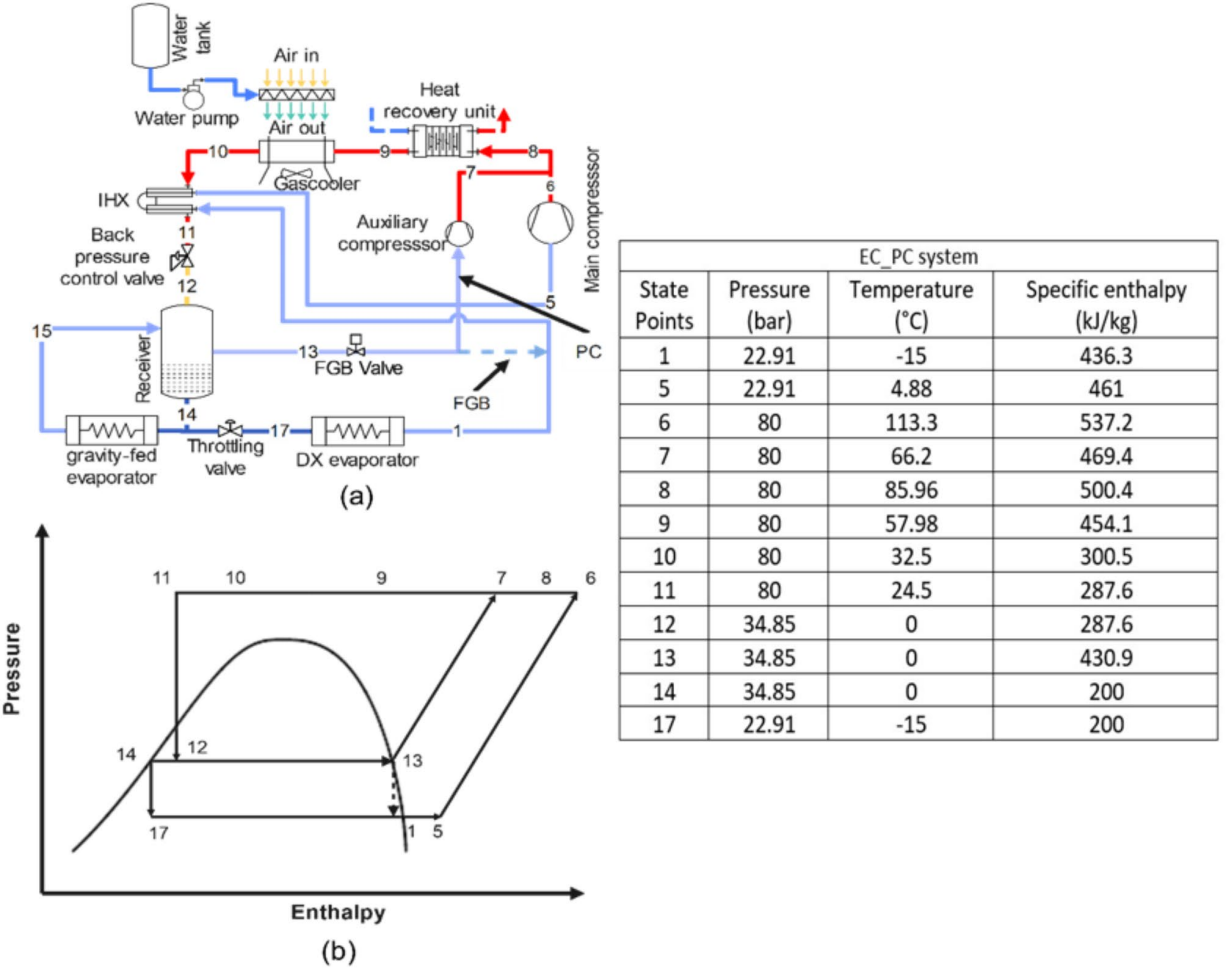
(**a**) Schematic of EC_fgb and EC_PC (**b**) corresponding p-h diagram. Table represents the state points according to P-h diagram of EC_PC system.

- **Base_fgb and Base_PC system**: The Base_fgb system is a dual evaporator configuration where the high-temperature evaporator operates gravity-fed without a throttling valve, maintained at receiver pressure, which is regulated by the FGB valve to meter flash gas flow. The low-temperature evaporator uses a DX configuration with throttling from the receiver pressure and includes a flash gas bypass valve and a heat recovery unit. In the Base_PC system, the Base_fgb setup is modified by replacing the FGB valve with a PC due to high flash gas generation (Fig. 1a, p-h diagram Fig. 1b).
- **DMS_fgb and DMS_PC system**: The DMS_fgb system is the Base_fgb system equipped with a DMS. In the DMS_PC system, the Base_PC system is equipped with a DMS, utilizing propane (R290) in the DMS cycle (Fig. 2a, p-h diagram Fig. 2b).
- **IMS_fgb system**: The IMS_fgb system is the Base_fgb system equipped with an IMS, where the refrigerant is split into two streams, with one subcooling the other before recompression. In the IMS_PC system, the Base_PC system is equipped with an IMS (Fig. 3a, p-h diagram Fig. 3b).
- **EC_fgb system**: The EC_fgb system is a modified version of the Base_fgb system, incorporating evaporative cooling at the gas cooler. Similarly, the EC_PC system modifies the Base_PC system by adding evaporative cooling at the gas cooler (Fig. 4a, p-h diagram Fig. 4b).

Further, the detailed schematic of all 8 configurations are given in the supplementary file in the Fig. S1 to Fig. S8 along with the corresponding p-h chart.

The modifications of technologies adopted for each of the eight configurations are summarized in (Table 1).

**Table 1:** Technologies adopted for each configuration.

	Configuration	FGB	PC	IMS	DMS	EC
1.	*Base_fgb*	✓	X	X	X	X
2.	*Base_PC*	X	✓	X	X	X
3.	*DMS_fgb*	✓	X	X	✓	X
4.	*DMS_PC*	X	✓	X	✓	X
5.	*IMS_fgb*	✓	X	✓	X	X
6.	*IMS_PC*	X	✓	✓	X	X
7.	*EC_fgb*	✓	X	X	X	✓
8.	*EC_PC*	X	✓	X	X	✓

*FGB* = flash gas bypass, *PC* = parallel compression, *IMS =* integrated mechanical subcooling, *DMS =* dedicated mechanical subcooling, *EC =* evaporative cooling.

**Fig. 5:**
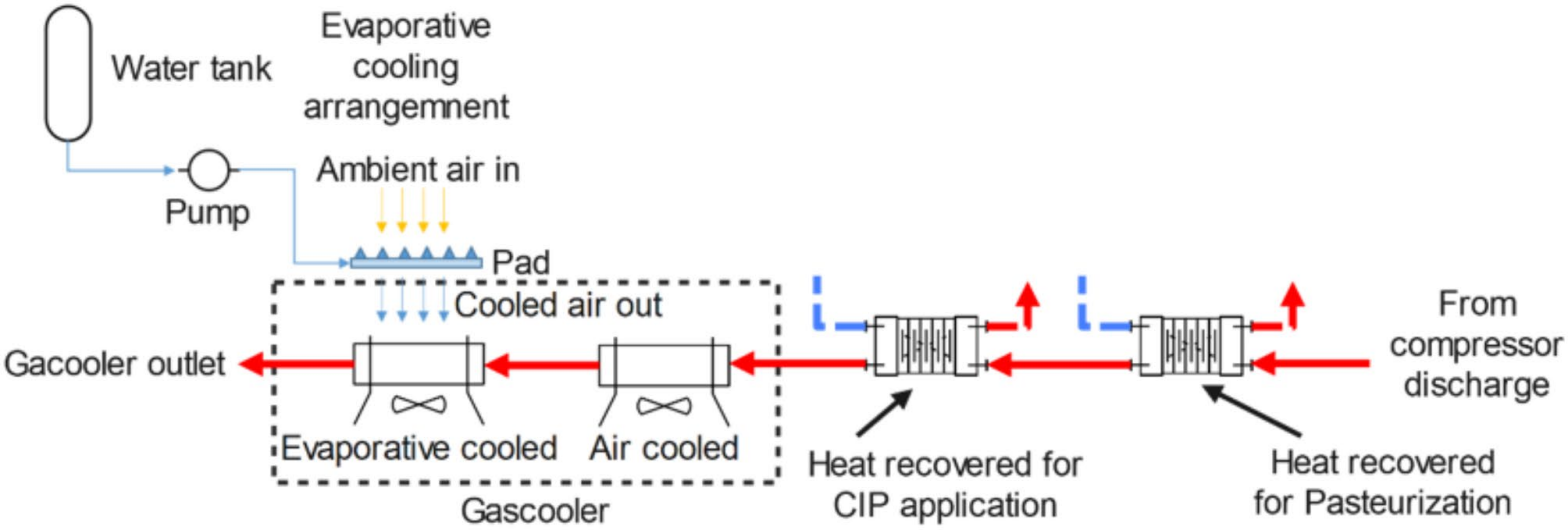
Potential modifications in the gascooler and heat recovery unit, along with proposed system.

The discharge temperature and heat recovery potential of all proposed systems were calculated and analyzed to assess their capability to fully meet the heating requirements for the milk pasteurization process, targeting a temperature of 72 °C and a heating capacity of 50 kW. Any excess heating capacity may be redirected to the CIP (clean-in-place) application through an auxiliary heat recovery heat exchanger (Fig. 5).

Additionally, the potential for reducing water consumption in evaporative cooling is explored by utilizing a split gas cooler, as detailed by Singha et al.^20^. In this setup, the first section of the gas cooler is air-cooled, while the second section is cooled using evaporative cooling.

### Mathematical modelling

The mathematical model for the proposed systems is formulated based on the following assumptions^21,22^.

- Steady state operation and Negligible pressure drop and heat loss across the components.
- Throttling process is isenthalpic.
- Negligible power consumed by the fan.
- Compression work is non-isentropic.

The $\:COP$ of the system is computed using Eq. (1)

1 $$\:COP=\:\frac{{\dot{Q}}_{eva}+\:{\dot{Q}}_{gfe}}{{\dot{W}}_{total}}$$

Where $\:COP$ measures the cooling performance of system. $\:{\dot{Q}}_{eva}\:$and $\:{\dot{Q}}_{gfe}$ are the cooling loads in the LT evaporator and gravity-fed evaporator respectively.

2 $$\:{\dot{W}}_{total}=\:{\dot{W}}_{main}+{\dot{W}}_{auxiliary}+{\dot{W}}_{DMS}+\:{\dot{W}}_{IMS}+\:{\dot{W}}_{pump}$$

$\:{\dot{W}}_{total\:}$includes work done by main compressor ($\:{\dot{W}}_{main}$), auxiliary compressor ($\:{\dot{W}}_{auxiliary}$), DMS compressor ($\:{\dot{W}}_{DMS}$), IMS compressor ($\:{\dot{W}}_{IMS}$) and the pump work input ($\:{\dot{W}}_{pump}$) for the evaporative cooling arrangementEach compressor and pump work input is calculated as given in Eq. (3)

3 $$\:{\dot{W}}_{comp/pump}=\:{\dot{m}}_{comp/pump}\:\times\:\varDelta\:h$$

Where $\:{\dot{m}}_{comp/pump}$ and $\:\varDelta\:h$ are the refrigerant mass flow rate and enthalpy difference across compressor/pump. The CO_2_ compressor isentropic efficiency ($\:{\eta\:}_{is,CO2}$) is calculated using the correlation^23^ given in Eq. (4), considering $\:{R}_{CO2}$ is the pressure ratio across each CO_2_ compressor

4 $$\:{\eta\:}_{is,\:CO2}=\:0.9343-0.04478\times\:{R}_{CO2}$$

The isentropic efficiency of the DMS compressor $\:{\eta\:}_{is,DMS}$ is given in Eq. 5 considering $\:{R}_{DMS}$ is the pressure ratio across DMS compressor

5 $$\:{\eta\:}_{is,\:DMS}=\:0.83955-0.01026\times\:{R}_{DMS}-0.00097\times\:{R}_{DMS}^{2}$$

Equations (4) and (5) denotes that $\:{\eta\:}_{is,\:DMS}$ and $\:{\eta\:}_{is,\:CO2}$ depends on the pressure ratio of the respective compressors. The cooling load on the subcooler heat exchanger $\:{Q}_{sub}$ is calculated using Eq. (6)

6 $$\:{Q}_{sub}=\:{\dot{m}}_{Sub}\times\:\varDelta\:{h}_{sub}$$

$\:{Q}_{sub}$ is mostly depends on the degree of subcooling. Here $\:{\dot{m}}_{Sub}$ and $\:\varDelta\:{h}_{sub}$ are the refrigerant mass flow rate and the enthalpy difference across the subcooler heat exchanger.

The heat recovery potential ($\:{\dot{Q}}_{recovered}$) is calculated using Eq. (7)

7 $$\:{\dot{Q}}_{recovered}=\:{\dot{m}}_{water}\:\times\:\varDelta\:{h}_{water}$$

The $\:{\dot{Q}}_{recovered}$ denotes the available heat on the higher side of the system which can be utilized. Where $\:{\dot{m}}_{water}$ and $\:\varDelta\:{h}_{water}$ are the secondary fluid side mass flow rate and enthalpy difference across heat recovery unit.

The combined coefficient of performance ($\:CCOP$) includes the cooling capacity and the heat recovery potential of the system $\:{\dot{Q}}_{recovered}$ as given by Eq. (8)

8 $$\:CCOP=\:\frac{{\dot{Q}}_{eva}+\:{\dot{Q}}_{gfe}+{\dot{Q}}_{recovered}}{{\dot{W}}_{total}}$$

$\:CCOP$ denotes the total output of the system considering both heating and cooling effects. The temperature of the air $\:{(t}_{air})\:$after evaporative cooling depends on the parameter like efficiency of mixing pad ($\:{\epsilon}_{mixing}$), dry bulb temperature (DBT) $\:{(t}_{dbt})$ and wet bulb temperature$\:\:\left(\text{W}\text{B}\text{T}\right)\:\:\left({t}_{wbt}\right)$. The same is given in Eq. (9)

9 $$\:{\epsilon}_{mixing}=\:\frac{{t}_{dbt}-\:{t}_{air}}{{t}_{dbt}-\:{t}_{wbt}}$$

The higher $\:{\epsilon}_{mixing}$ denotes enhanced heat and mass transfer, The higher $\:{\epsilon}_{mixing}$ denotes higher heat and mass transfer. $\:{t}_{wbt}$ is a function of $\:{t}_{dbt}$ and dew point temperature $\:\left({t}_{dew}\right)$ as given in Eq. (10)

10 $$\:{t}_{wbt}=\:f({t}_{dbt},\:{t}_{dew})$$

The heat rejected by the evaporative cooled gascooler ($\:{Q}_{GC\_eva}$) is calculated as given in Eq. (11), where $\:{\dot{m}}_{ref\_gc}$ and $\:\varDelta\:h$ are the refrigerant mass flow rate and enthalpy difference respectively.

11 $$\:{Q}_{GC\_eva}=\:{\dot{m}}_{ref\_gc}\times\:\varDelta\:h$$

The mass of moist air ($\:{\dot{m}}_{moist\:air}$) passed through the evaporative gascooler is calculated as given in Eq. (12) where $\:\varDelta\:{h}_{air}$ is the enthalpy difference of the inlet and outlet air from the gascooler respectively

12 $$\:{M}_{moist\:air}=\:\frac{{Q}_{GC\_eva}\:}{\varDelta\:{h}_{air}}$$

The total water consumption is calculated by Eq. (13) where $\:{m}_{water}$ denotes the water consumption

13 $$\:{M}_{water}=\:{\dot{m}}_{moist\:air}\:\times\:\varDelta\:\omega\:$$

Where $\:\varDelta\:\omega\:$ is the difference between humidity ratio of the air before and after evaporative cooling.

The boundary conditions for simulation to evaluate the performance of the systems are given in (Table 2). The simulation is carried out in EES software^24^ and the in-built library of refrigerant properties are used.

**Table 2:** Input parameters for the simulation.

Parameters	Values
DX evaporator cooling capacity	150 kW
DX evaporator temperature	−15 °C
gravity-fed evaporator cooling capacity	50 kW
gravity-fed evaporator temperature	0 °C
Average ambient temperature	35 °C
Average dew point temperature	20 °C
IHX effectiveness	0.5
Degree of subcooling for IMS and DMS	1–5 K
Gas cooler approach temperature	5 K

### Model validation

**Fig. 6:**
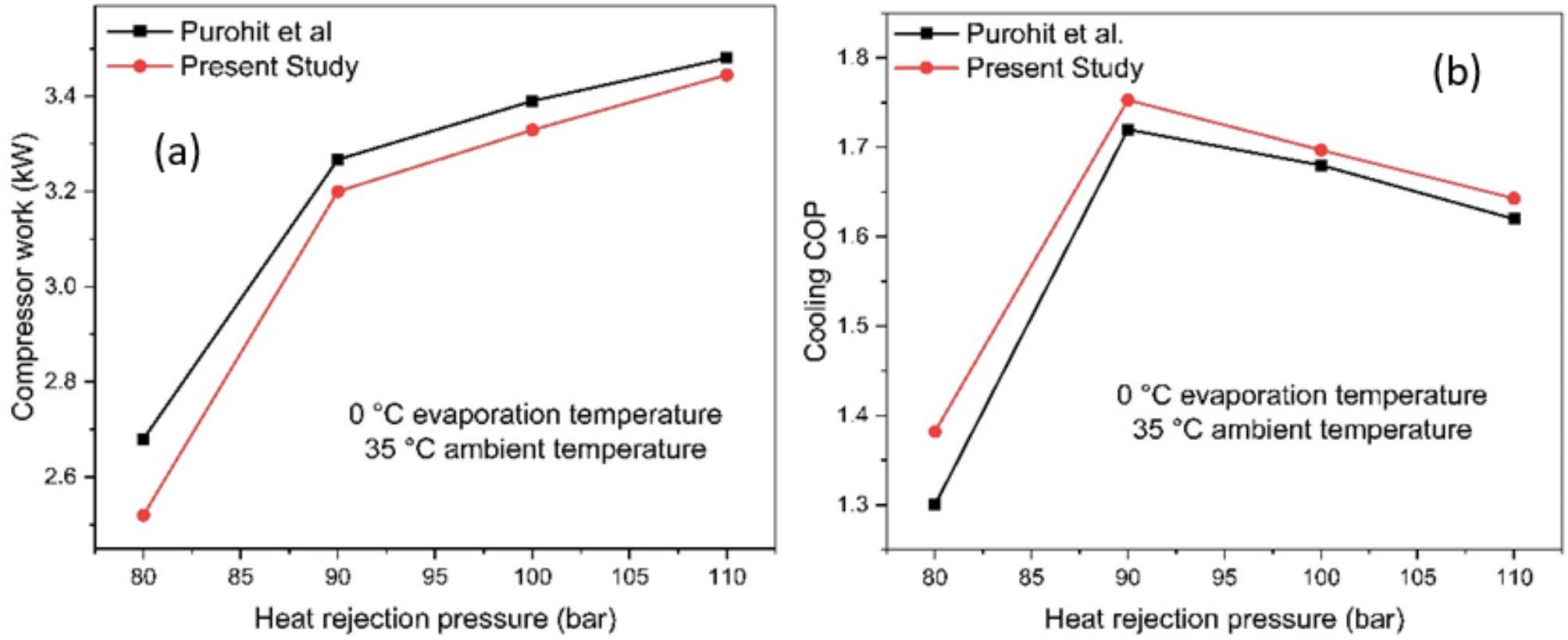
Validation of the model (**a**) Compressor work and (**b**) COP.

The thermodynamic model of the Base_fgb is validated against the study by Purohit et al.^25^. The system is simulated at a constant ambient temperature of 35 °C and an evaporation temperature of 0 °C, with heat rejection pressure varied from 80 bar to 110 bar. The performance metrics (compressor work and COP) are compared in (Fig. 6a,b). The average and maximum deviations for compressor work are 2.64 and 6.0%, respectively, while for COP, the average and maximum deviations are 2.61 and 6.3%. Further, the deviation of the current study with Purohit et al.^25^ is tabulated in (Table 3).

**Table 3:** Validation of the present study with Purohit et al.^25^.

Evaporation temperature (°C)	Ambient temperature (°C)	Heat rejection pressure (bar)	Compressor work		COP		% deviation (Compressor work)	% deviation (COP)
			Present study	Purohit et al.^25^	Present study	Purohit et al.^25^		
0	35	80	2.52	2.68	1.38	1.30	6.3	6.0
0	35	90	3.21	3.27	1.75	1.72	1.8	1.8
0	35	100	3.33	3.39	1.697	1.68	1.2	1.8
0	35	110	3.44	3.48	1.64	1.62	1.1	1.0

## Results and discussion

The present study explores the performance of a dual evaporator transcritical CO_2_ system for milk processing application. Two different subcooling techniques, DMS and IMS are explored. For all thermodynamic analyses, the average weather conditions of Pune, India are considered. The performance of all proposed systems, detailed in Table 1, is evaluated at the optimal heat rejection pressure using the built-in functionality of EES, and then compared with the Base_fgb system.

**Fig. 7:**
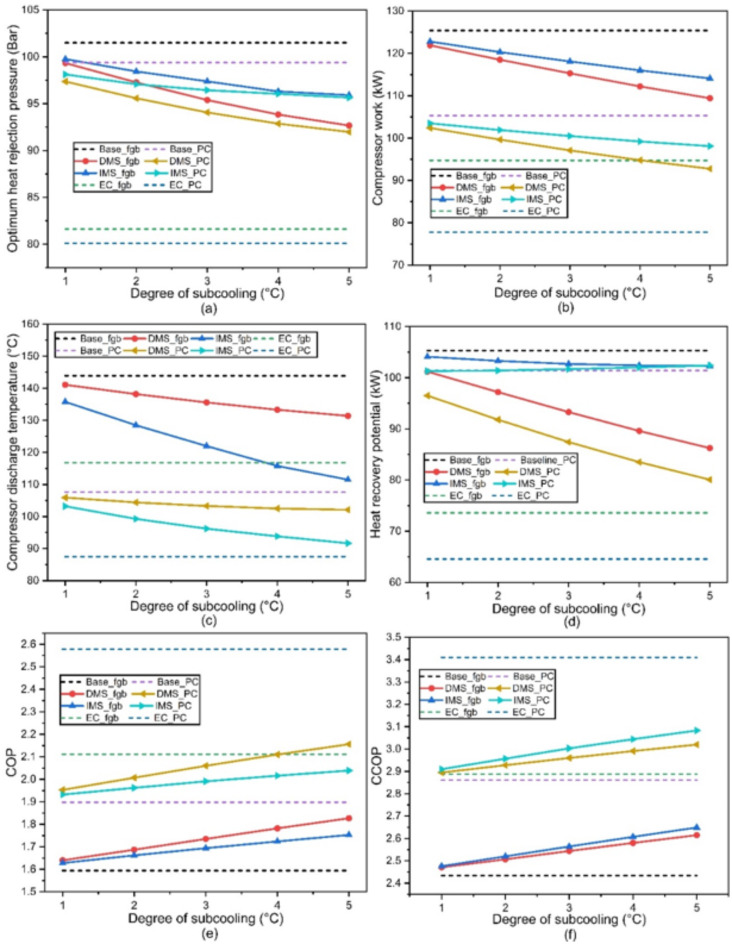
Parameters varying with for different configurations: (**a**) optimum heat rejection pressure, (**b**) compressor work, (**c**) discharge temperature, (**d**) heat recovery potential, (**e**) COP and (**f**) CCOP.

Parameters such as optimum heat rejection pressure ($\:{P}_{g}$) (Fig. 7a), compressor work (Fig. 7b), discharge temperature (Fig. 7c), heat recovery potential (Fig. 7d), COP (Fig. 7e) and CCOP (Fig. 7f) of the proposed systems (Table 1) are evaluated and compared with the Base_fgb configuration. For all the configurations, the heat rejection pressure is optimized for the maximum COP^26^. The degree of subcooling (DOS) is varied from 1 to 5 K for systems equipped with DMS (DMS_fgb and DMS_PC) and IMS (IMS_fgb and IMS_PC) while the other systems (Base_fgb, Base_PC, EC_fgb and EC_PC) do not have subcooling arrangement. The cooling load for DMS and IMS equipped configurations depends on the DOS while the subcooling provided by the IHX depends on the effectiveness of IHX.

The $\:{P}_{g}$ of Base_fgb system is found 101.5 bar which is the highest among all the configurations. The higher $\:{P}_{g}$ results in higher compressor work with a value of 125.4 kW, this is because of the higher compression ratio and higher refrigeration mass flow rate resulting in in higher compressor work. The higher $\:{P}_{g}$ directly influences the compressor discharge temperature with a value of 144 °C. The higher discharge temperature and higher refrigerant mass flow rate results in higher heat recovery potential of the system, leading to the value of 105.3 kW. The higher compressor work results in a lower COP of the system. However, the higher heat recovery potential of the system leads to a comparably higher CCOP. The COP and CCOP of the system is found 1.53 and 2.43 respectively.

For Base_PC system, parallel compression is adopted in Base_fgb system instead of FGB. The heat rejection pressure for this Base_PC system is decreased to 99.4 bar, resulting in a decrement of 2.06%. Due to the reduced$\:{\:P}_{g}$, the compressor work is reduced around 16%, also reducing compressor discharge temperature to 108 °C. The reduced discharge temperature and $\:{P}_{g}$ leads to a lower heat recovery potential. The heat recovery potential of the Base_PC reduced about 3.7% .The COP of Base_PC is improved about 19.1% while the CCOP of the system 17.5%. This attributed to the fact that the effect of COP improvement is more dominant compared to the reduction of the heat recovery potential.

Following that, DMS (DOS, 1–5 K) is adopted with Base_fgb and Base_PC configurations and denoted as DMS_fgb and DMS_PC respectively. It is noticed that the $\:{P}_{g}$ decreases with increase in DOS. For DMS_fgb configuration, $\:{P}_{g}$ decreases by 2.1–8.7%, resulting in a reduction in compressor work by 2.8–12.8%. This attributed to the fact that decreased $\:{P}_{g}$ and improvement in specific cooling capacity leads to reduced refrigerant mass flow rate, resulting in lower compressor work. It is also observed that the discharge temperature decreases as DOS increases. For the DMS_fgb configuration, the discharge temperature decreases from 141 °C to 131 °C. Due to the reduced discharge temperature and reduced refrigeration mass flow rate, the heat recovery potential is found to be decreasing from 3.9 to 18.1% when compared to Base_fgb system. Further the COP and CCOP is found to be increasing with increment in DOS. This is attributed to the fact that reduced compressor work leads to an improved COP. The CCOP improvement is due to the greater impact in COP than the reduction in heat recovery potential. The COP is found to be increased by 2.9 to 14.6% while the CCOP improvement is from 1.5 to 7.4%. For DMS_PC configuration, $\:{P}_{g}\:$is found to be decreasing further. This is due to the fact that auxiliary compressor compresses the flash gas over a lower pressure ratio (receiver pressure to heat rejection pressure). For DMS_PC configuration, $\:{P}_{g}$ found to decrease by 4.1–9.4%, leading a reduced compressor work by 18.3–26%. The discharge temperature is decreases to 106 °C–102 °C. The heat recovery potential is further decreased due to reduction in $\:{P}_{g}$ and found to decrease by 8.4–24%. The COP is found to be improving by 22.5–35.2% while the CCOP is improving from 18.9 to 24%.

Further, IMS is adopted with Base_fgb and Base_PC configurations and termed as IMS_fgb and IMS_PC respectively. Similar to DMS systems, it is observed that $\:{P}_{g}$ decreases in IMS with an increase in DOS. Due to the higher$\:{\:P}_{g}$, the compressor work for IMS_fgb system is higher compared to the DMS_fgb system. For the IMS_fgb configuration,$\:{\:P}_{g}$ decreases by 1.7–5.5%, leading to a reduction in compressor work by 2.1–9%.

As expected, the discharge temperature is found decreasing with DOS. The discharge temperature is decreasing from 136 °C to 111 °C. Further the rate of decrement in discharge temperature is noticed higher compared to DMS_fgb system for higher DOS. It is worth noticing that although the $\:{P}_{g}$ is higher in case of IMS_fgb system, discharge temperature of the system is lower compared to DMS_fgb system. This is due to the lower discharge temperature of the subcooler compressor, which reduces the temperature at the compressor discharge after mixing state point 8 in (Fig. 3a). The heat recovery of IMS_fgb system is explored and found decreasing with DOS with a value of 1.2–2.9% when compared with Base_fgb system. It is interesting to notice that despite of having lower discharge temperature, the heat recovery in IMS_fgb is found higher compared to DMS_fgb system. This is attributed to the increased refrigerant mass flow rate through the heat recovery unit, resulting from the additional refrigerant flowing through the subcooler compressor. The improvement in COP and CCOP of IMS_fgb is found around 2.1–9.09% and 1.7− 8.8% respectively. Further when parallel compression is adopted (IMS_PC), it is observed that $\:{P}_{g}$ decreases with an increase in DOS, but the rate of this decrease diminishes at higher DOS values. This is because, at higher DOS, the refrigerant properties approach the pseudocritical zone. Comparing with Base_fgb system, the reduction in $\:{P}_{g}$ is found around 3.3–5.8%. The reduced $\:{P}_{g}$ results 17.5–21.8% reduction in compressor work when compared with Base_fgb system. As expected the discharge temperature is decreasing from 103 °C to 92 °C. As explained in the IMS_fgb configuration, the discharge temperature of IMS_PC is lower compared to the DMS_PC system. Interestingly, it is found that the heat recovery potential of IMS_PC increases with increase in DOS. This is attributed to the fact that the refrigerant approaches to its pseudocritical zone which further increases the refrigerant mass flow rate through the subcooling heat exchanger. Therefore, the quantity of the heat recovered is increased. For IMS_PC configuration, the heat recovery potential varies from 101.3 to 102.4 kW, a reduction of 3.8–2.7% when compared with Base_fgb system. Further the COP and CCOP of the IMS_PC is computed and found an improvement of 21.2–27.9% and 19.5–36.5% respectively.

Further, evaporative cooling arrangement to the gascooler is also explored considering the average ambient temperature of Pune, India. The Base_fgb and Base_PC are equipped with evaporative cooling arrangement and termed as EC_fgb and EC_PC. It is found that, with evaporative cooling arrangement, the temperature of the inlet air to the gascooler can be reduced by about 7.5 °C to 27.5 °C which reduces the $\:{P}_{g}$ around 19.6%, the lowest among all the configurations with flash gas bypass, and improves the specific cooling capacity. The reduced $\:{P}_{g}$ leads to a decrement of 24.5% in compressor work when compared to Base_fgb system. The reduced $\:{P}_{g}$ results in lower discharge temperature of 116.8 °C and the heat recovery potential of the system reduces by 30.1%. The reduction in compressor work results in substantial improvement in COP of about 32.4%. Despite of having significant decrement in heat recovery potential, the CCOP improvement is also substantial as the improvement in COP is more dominant. An improvement of about 18.6% is found in CCOP. Further when the parallel compression is adopted, a marginal decrement in $\:{P}_{g}$ of 21.2% is found when compared to Base_fgb system. Due to the reduced $\:{P}_{g}\:$a reduction of 38% is found in compressor work, which is the lowest among all the configuration. Further the compressor discharge temperature of 88 °C is found, which is the lowest among all the configurations. Due to the lowest discharge temperature and$\:{\:P}_{g}$, the heat recovery potential of the system is found the lowest, which is 38.7% lower compared to Base_fgb system.

A comparing the compressor work for systems utilizing FGB and PC technologies is summarised. It is observed that the Base_fgb system requires 125 kW of work, which decreases to 105.3 kW, representing a 16% reduction. Similarly, for the DMS_fgb system, as the degree of subcooling (DOS) increases from 1 K to 5 K, the compressor work reduces from 121.9 kW to 109.4 kW. Under the same conditions, it decreases further from 102.4 kW to 92.75 kW. For the IMS_fgb system, the compressor work drops from 122.8 kW to 114.1 kW as DOS increases from 1 K to 5 K, with a corresponding reduction from 103.5 kW to 98.1 kW. The inclusion of evaporative cooling significantly reduces compressor work, with the EC_fgb configuration requiring 94.72 kW, and the EC_PC configuration further decreasing to 77.78 kW. The compressor work ($\:\varDelta\:{W}_{comp}$) and COP ($\:\varDelta\:COP$) of all the PC equipped systems are compared with FGB equipped system and the same is tabulated in (Table 4). Further, the performance parameters of all the systems are tabulated in Table. TS1 of the supplementary file.

**Table 4:** Comparison of compressor work and COP of PC and FGB equipped systems.

	Base_fgb and Base_PC system (%)	DMS_fgb and DMS_PC system (%)	IMS_fgb and IMS_PC system (%)	EC_fgb and EC_PC system (%)
$\:\varDelta\:{W}_{comp}$	16.03	15.99–15.22	15.71− 14.02	17.88
$\:\varDelta\:COP$	19.1	16.02–15.26	15.73–14.03	22.12

The EC_PC system exhibits the lowest discharge temperature and heat recovery potential among all proposed systems, with values of 87.5 °C and 64.5 kW, respectively. This is adequate to fully meet the heating requirements for the milk pasteurization process, which targets a temperature of 72 °C and a heating capacity of 50 kW. Any excess heating potential can be utilized for the CIP (clean in place) process using an additional heat recovery heat exchanger, as shown in (Fig. 5).

**Fig. 8:**
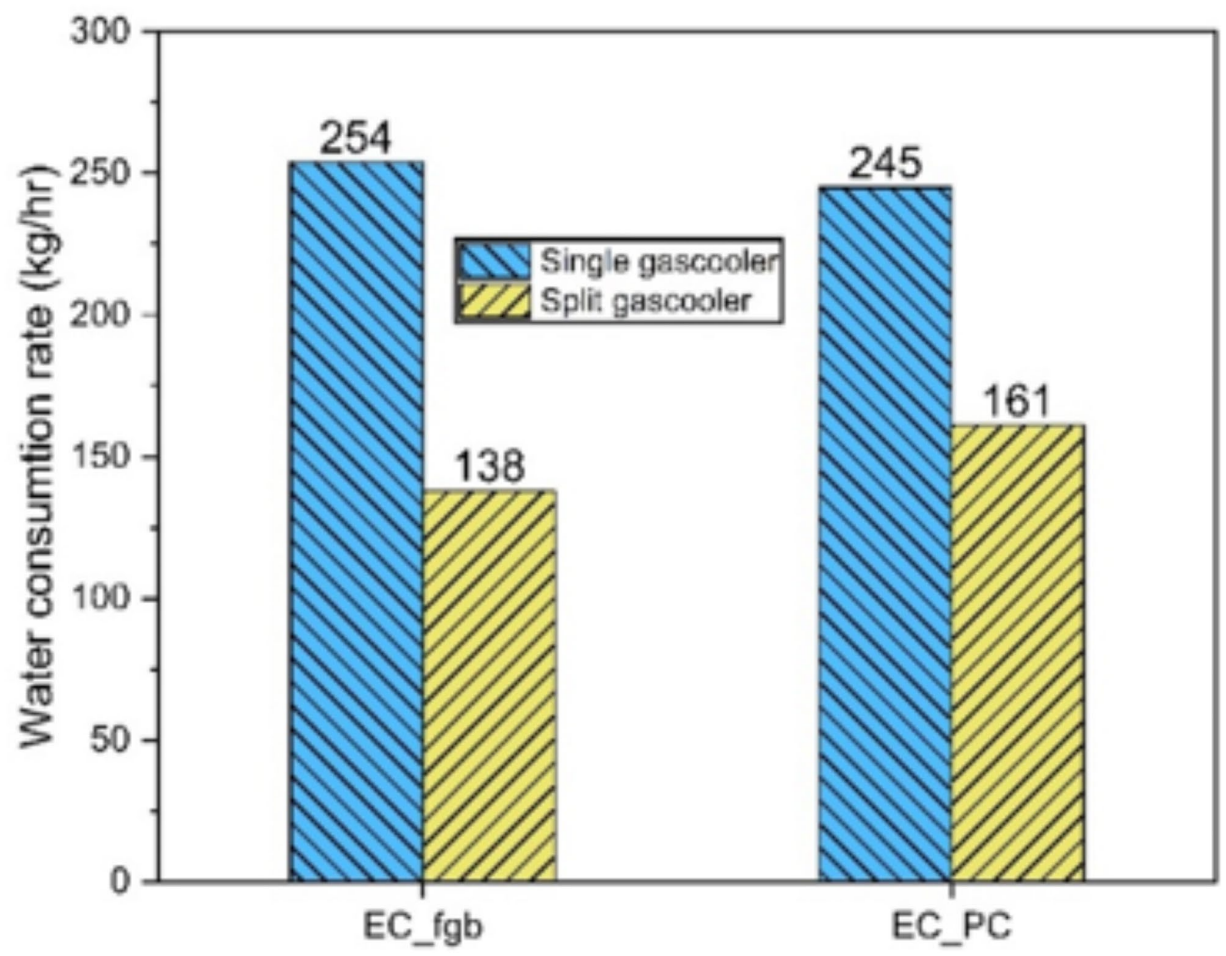
Change of water consumption by adopting split gascooler.

Furthermore, the split gas cooler features an air-cooled first section and an evaporative cooling second section. This setup reduces the water consumption of the EC_fgb system lower from 254 to 138 kg/hr, achieving a 45% reduction. In contrast, the EC_PC system (Fig. 8) shows a smaller water savings of 34.3% due to a lower total heat removal requirement, operating at a lower $\:{P}_{g}$ and resulting in decreased heat rejection by the air-cooled portion of the gas cooler.

The split gascooler system is particularly applicable in regions where availability of water in terms of quality and/or quantity—poses a challenge. This approach significantly reduces water consumption, with savings directly proportional to the mass flow rate of pre-cooled air which in turn, depends on the heat rejected by the evaporatively-cooled gascooler. However, to further minimize water usage, the heat rejection by the evaporatively-cooled gascooler must be reduced, which results in an increased heat rejection load on the air-cooled gascooler. Achieving this requires lowering the approach temperature of the air-cooled gascooler, a goal that leads to oversizing of the gascooler. In that case, the power input to the fan will increase which may conversely affect the system performance^27^.

## Conclusion

This study explores the performance improvement possibilities of a multi-evaporator transcritical CO_2_ systems for a milk processing application deploying a DX evaporator (−15 °C) and a gravity-fed evaporator (0 °C). Performance parameters for both heating and cooling application are explored, auxiliary compressor is used in place of flash gas bypass because of the high flash gas generation in the receiver due to the gravity-fed evaporator. Further, COP improvement strategies such as DMS, IMS and evaporative cooling arrangement to the gascooler are explored using eight different configurations, leading to the following findings.

- In all the configurations (Base, DMS, IMS, EC), incorporation of parallel compression improves the performance when gravity-fed evaporator is used.
- The maximum improvement in COP of 62.3% is observed in evaporative cooling with parallel compression due to the lowest heat rejection pressure and higher refrigeration effect, leading to the largest reduction in compressor work. The maximum reduction in heat recovery potential (38.7%) is also found for the same configuration.
- The maximum improvement of 40.3% in CCOP observed for parallel compression and evaporative cooling even though the heat recovery prospect diminishes.
- EC_PC configuration leads to the highest COP and least heat recovery potential while the Base_fgb system leads to the lowest COP and highest heat recovery potential.
- DMS_PC configuration is found to be the optimum solution where simultaneous heating and cooling is required as a service, like for milk processing.
- There is a possibility of reduction of water consumption in evaporative cooler using a split configuration. Water consumption can be reduced by 45.6% for EC_fgb and 34.3% for EC_PC configuration at (DBT 35 °C, DPT 20 °C).

Although the EC_PC configuration results in lowest heat recovery potential, the reduction in compressor work also reduces the compressor size and power input, both of which leads to lower component cost. Further, the reduced refrigeration mass flow rate and heat rejection pressure reduces pipe size, saves cost and enhances the compactness of the system.

## Electronic supplementary material

Below is the link to the electronic supplementary material.

Supplementary Material 1

## Data Availability

Data will be made available on request. For access, please contact the corresponding author at prosenjit.singha@pilani.bits-pilani.ac.in.

## List of symbols

CCOP: Combined coefficient of performance
CFC: Chlorofluorocarbon
CIP: Clean-in-place
COP: Coefficient of performance
DBT: Dry bulb temperature (°C)
DMS: Dedicated mechanical subcooling
DOS: Degree of subcooling (K)
DX: Direct expansion
EC: Evaporative cooling
FGB: Flash gas bypass
GWP: Global warming potential
h: Specific enthalpy (kJ/kg)
HCFC: Hydrochlorofluorocarbon
HFC: Hydrofluorocarbon
HFO: Hydrofluoroolefin
hr: Hour
IHX: Internal heat exchanger
P: Heat rejection pressure (Pa)
IMS: Integrated mechanical subcooling
M, m: Mass flow rate (kg/sec)
MP: Montreal protocol
ODP: Ozone depletion potential
PC: Parallel compression
PFAS: Per- and polyfluoroalkyl substances
R: Pressure ratio
$\:Q$: Evaporator cooling load (kW)
*t*: Temperature (°C)
$\:W$: Compressor work input (kW)
WBT: Wet bulb temperature (°C)
*Base_fgb*: Baseline system with FGB
*Base_PC*: Baseline system with PC
*DMS_fgb*: System with DMS and FGB
*DMS_PC*: System with DMS and PC
*EC_fgb*: System with EC and FGB
*IMS_fgb*: System with IMS and FGB
*IMS_PC*: System with IMS and PC
*EC_PC*: System with EC and PC

## Greek symbols

$\epsilon$: Mixing pad efficiency
$$\:\eta\:$$: Isentropic efficiency
$$\:\omega\:$$: Humidity ratio

## Subscript

*auxiliary*: Auxiliary compressor
*eva*: Evaporator
*dew*: Dew point temperature
*gfe*: Gravity-fed evaporator
*main*: Main compressor
*recovered*: Heat recovery potential
*ref_gc*: Refrigerant through gascooler
*sub*: Subcooler
*total*: Total compressor work
