# Supplementary material for: New approach to improve COP and heat recovery in transcritical CO2 refrigeration system for milk processing application
Source: Sci Rep. 2025 Feb 14;15:5502. doi: 10.1038/s41598-025-90067-3 (PMC11829025; doi:10.1038/s41598-025-90067-3)
Supplement: Supplementary file 1 — Supplementary Material 1 [file 41598_2025_90067_MOESM1_ESM.docx]

- **Base_fgb System**: A dual evaporator system where the high-temperature evaporator is gravity-fed without a throttling valve, maintaining receiver pressure. The low-temperature evaporator uses DX configuration, with throttling from receiver pressure. It includes a flash gas bypass valve and a heat recovery unit (Fig. S 1(a), p-h diagram Fig. S 1(b)).
- **Base_PC System**: The Base_fgb system is modified by replacing the FGB with a PC due to high flash gas generation (Fig. S 2(a), p-h diagram Fig. S 2(b)).
- **DMS_fgb System**: The Base_fgb system is equipped with a DMS (Fig. S 3(a), p-h diagram Fig. S 3(b)).
- **DMS_PC System**: The Base_PC system is equipped with a DMS (Fig. S 4(a), p-h diagram Fig. S 4(b)). Propane (R290) is used in the DMS cycle.
- **IMS_fgb System**: The Base_fgb system is equipped with an IMS (Fig. S 5(a), p-h diagram Fig. S 5(b)). In IMS, the refrigerant is split into two streams, with one stream subcooling the other before being recompressed.
- **IMS_PC System**: The Base_PC system is equipped with an IMS (Fig. S 6(a), p-h diagram Fig. S 6(b)).
- **EC_fgb System**: The Base_fgb system is modified with evaporative cooling at the gascooler (Fig. S7 (a), p-h diagram Fig. S7 (b)).
- **EC_PC System**: The Base_PC system is modified with evaporative cooling at the gascooler (Fig. S8 (a), p-h diagram Fig. S8 (b)).


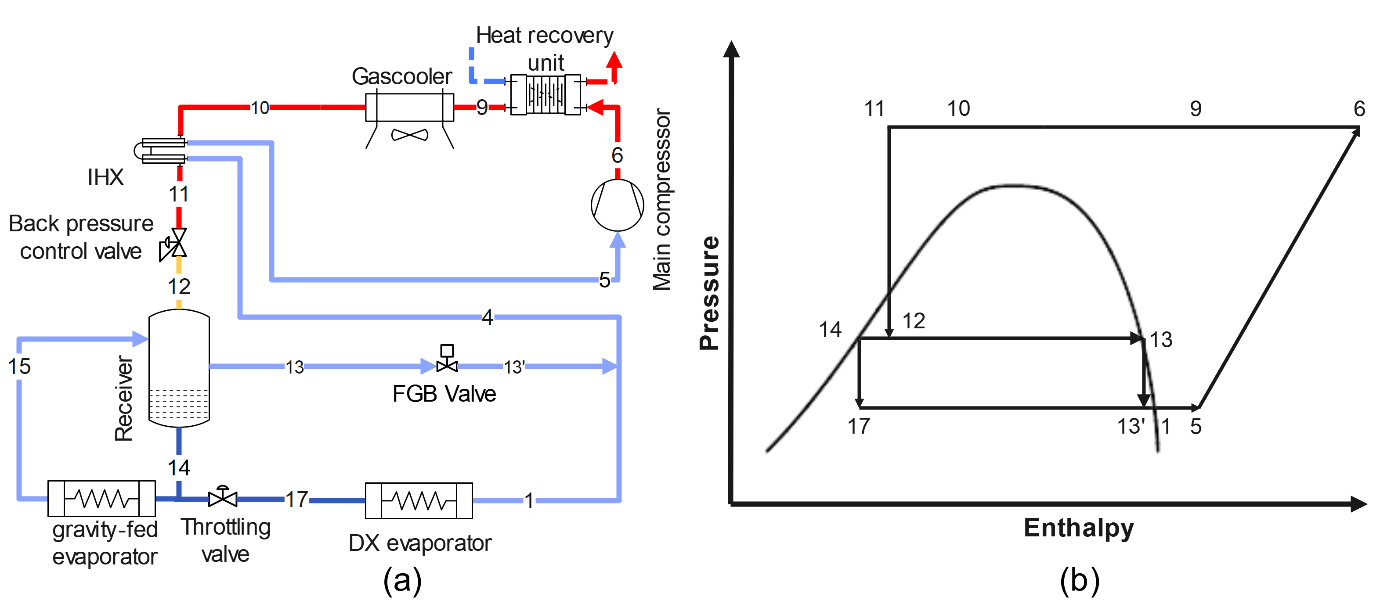


Fig. S1: (a) Schematic of Base_fgb system (b) corresponding p-h diagram


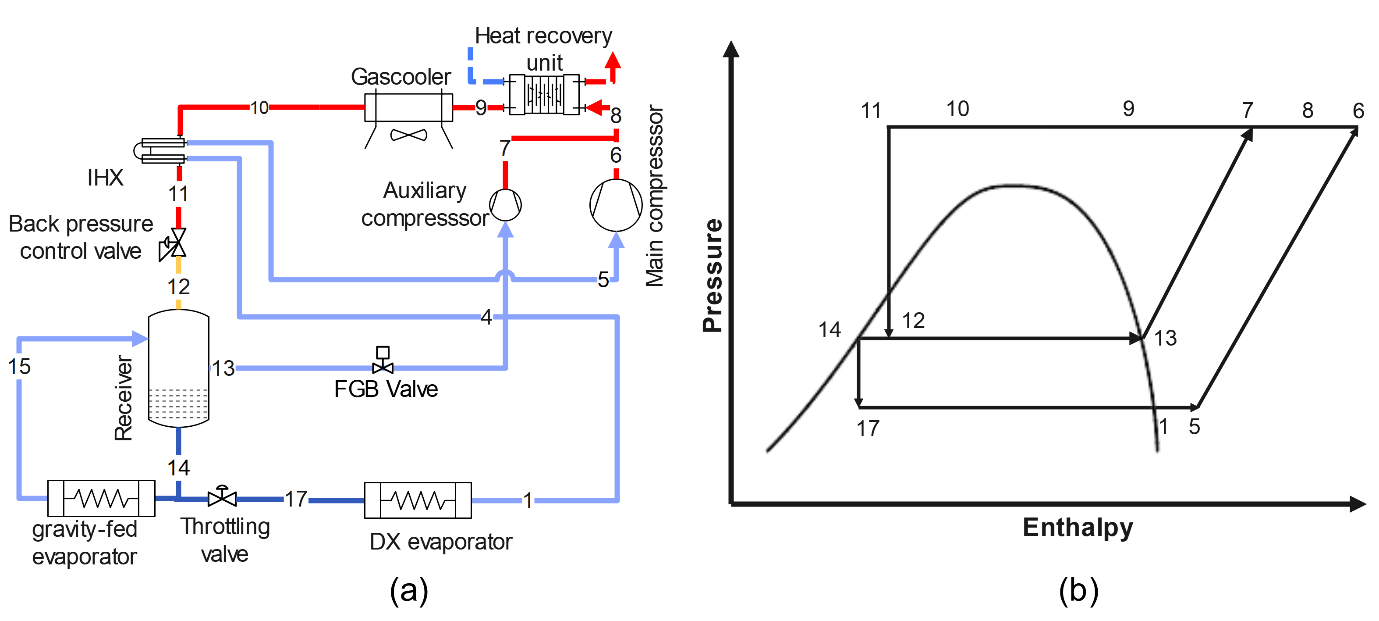


Fig. S2: (a) Schematic of Base_PC system (b) corresponding p-h diagram


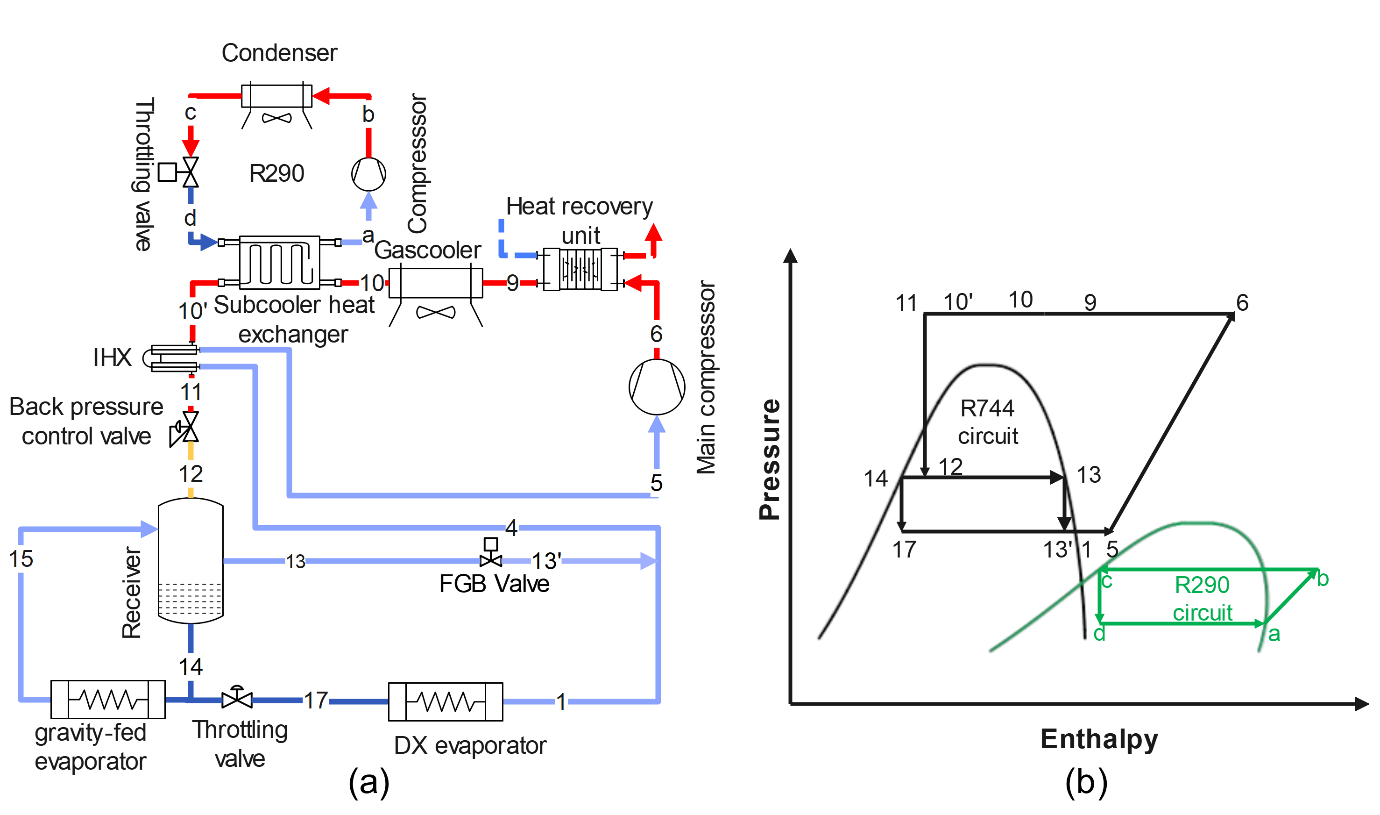


Fig. S3: (a) Schematic of DMS_fgb system (b) corresponding p-h diagram


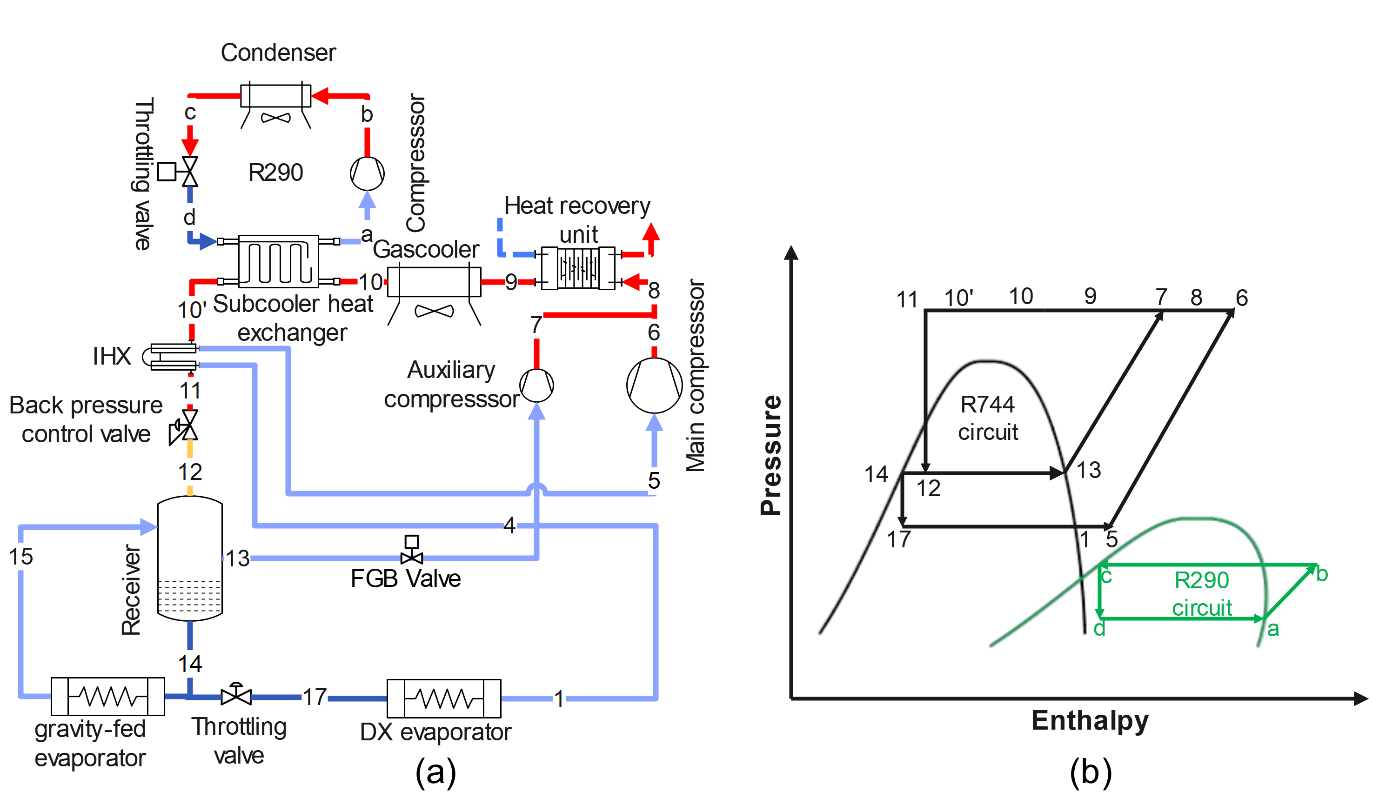


Fig. S4: (a) Schematic of DMS_PC system (b) corresponding p-h diagram


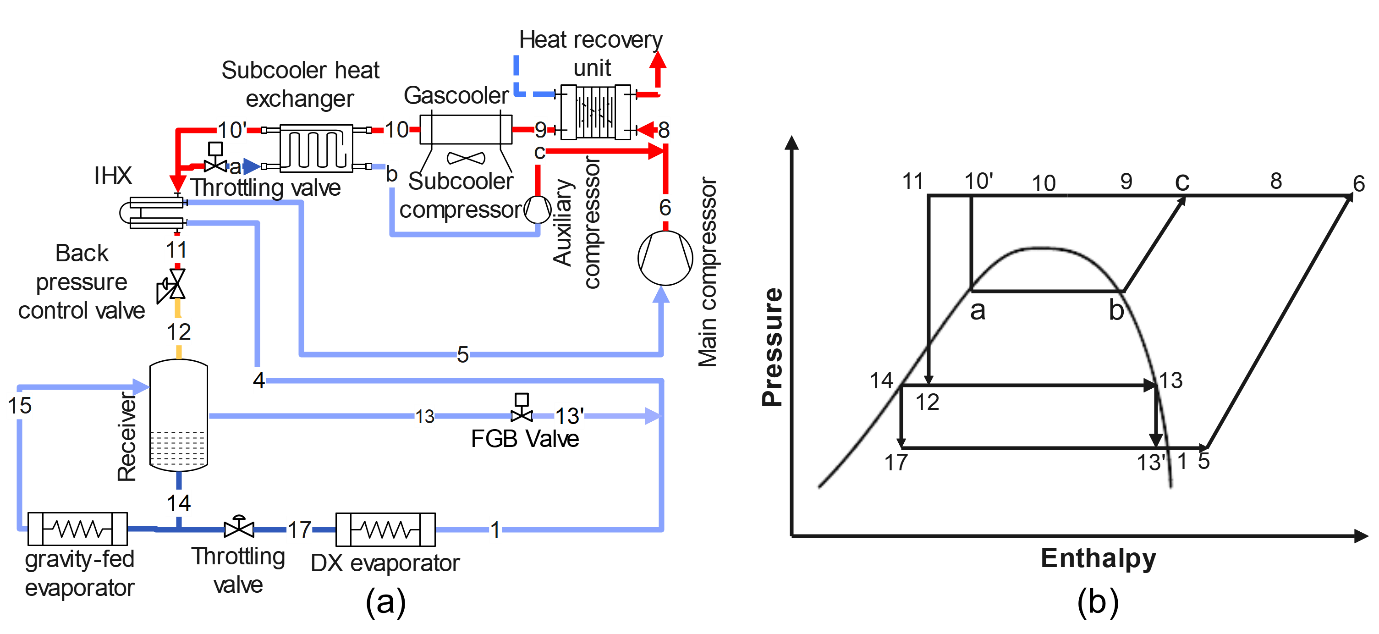


Fig. S5: (a) Schematic of IMS_fgb system (b) corresponding p-h diagram


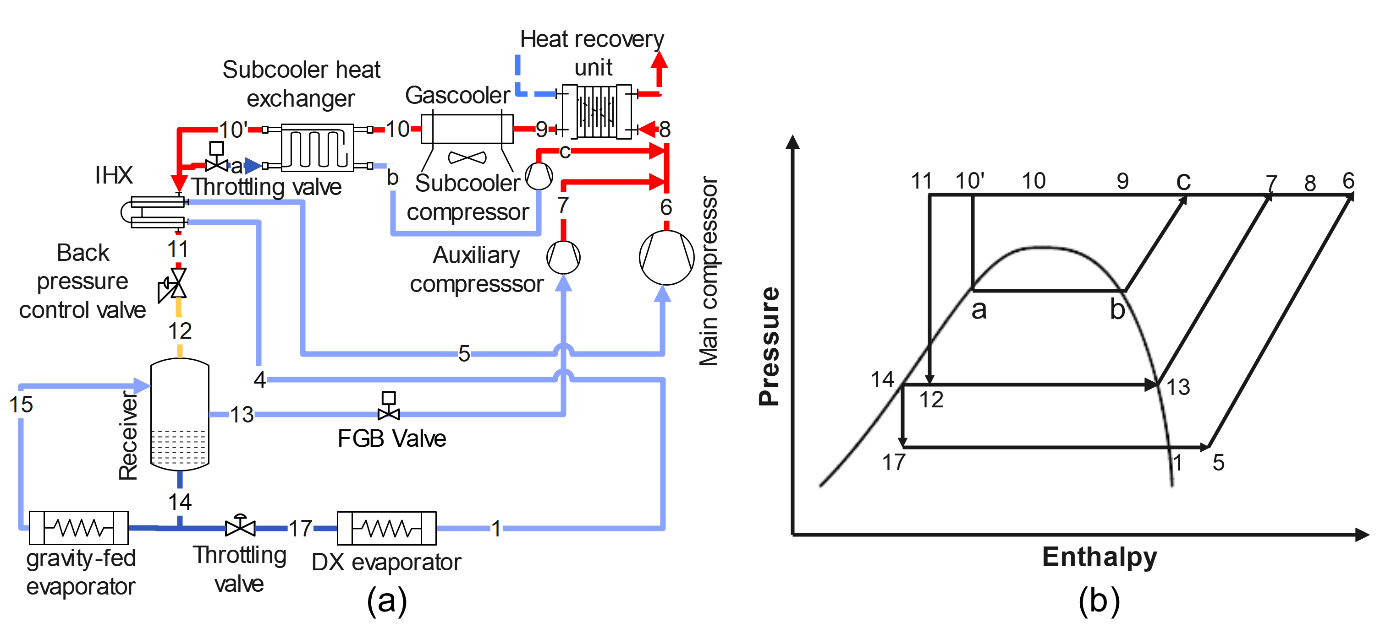


Fig. S6: (a) Schematic of IMS_PC system (b) corresponding p-h diagram


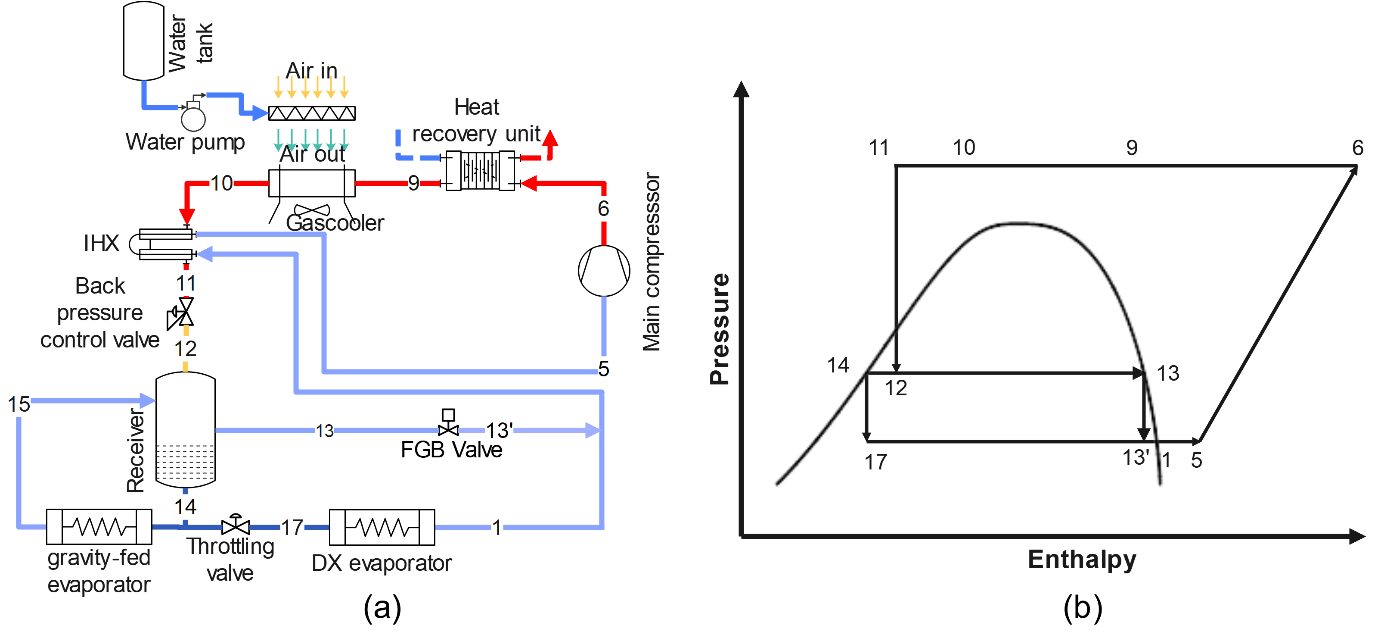


Fig. S7: (a) Schematic of EC_fgb system (b) corresponding p-h diagram


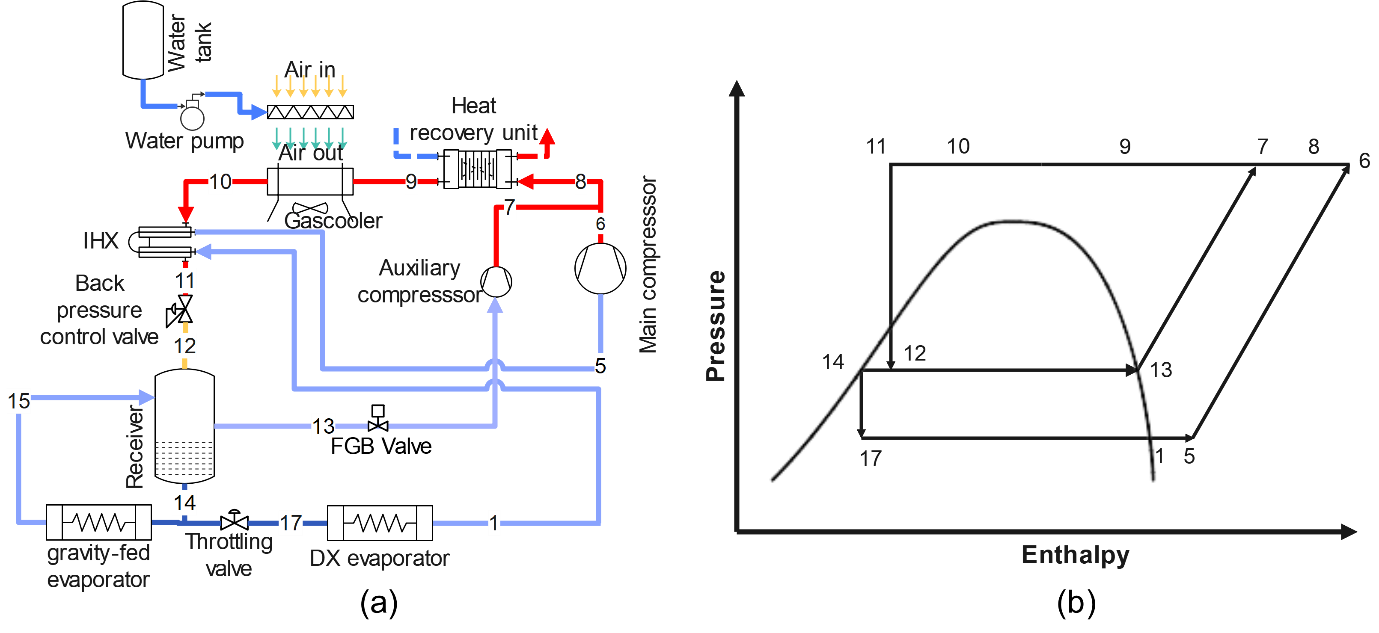


Fig. S8: (a) Schematic of EC_PC system (b) corresponding p-h diagram

Table TS1: Performance parameters of the proposed systems (Summarised in the table)

|  | Parameters | | | | | |
| --- | --- | --- | --- | --- | --- | --- |
| Systems | Optimum heat rejection pressure (bar) | Compressor work (kW) | Discharge temperature (°C) | Heat recovery potential (kW) | COP | CCOP |
| ***Base_fgb*** | 101.5 | 125.4 | 144 | 105.3 | 1.59 | 2.43 |
| ***Base_PC*** | 99.4 | 105.3 | 108 | 101.4 | 1.9 | 2.86 |
| ***DMS_fgb*** | 99.33 – 92.67 | 121.9 – 109.4 | 141 - 131 | 101.2 – 86.24 | 1.64 – 1.83 | 2.47 – 2.62 |
| ***DMS_PC*** | 97.4 – 92 | 102.4 – 92.7 | 106 - 102 | 96.5 - 80 | 1.95 – 2.16 | 2.89 – 3.02 |
| ***IMS_fgb*** | 99.76 – 95.91 | 122.8 – 114.3 | 136 – 111 | 104.1 – 102.3 | 1.63 – 1.75 | 2.48 – 2.65 |
| ***IMS_PC*** | 98.12 – 95.65 | 103.5 – 98.1 | 103 – 91.6 | 101.3 – 102.4 | 1.93 – 2.04 | 2.91 – 3.08 |
| ***EC_fgb*** | 81.62 | 94.72 | 117 | 73.58 | 2.11 | 2.89 |
| ***EC_PC*** | 80 | 77.8 | 87.5 | 64.55 | 2.58 | 3.41 |
